# Influence of glioma tumour microenvironment on the transport of ANG1005 via low-density lipoprotein receptor-related protein 1

**DOI:** 10.1038/bjc.2011.427

**Published:** 2011-10-25

**Authors:** Y Bertrand, J-C Currie, J Poirier, M Demeule, A Abulrob, D Fatehi, D Stanimirovic, H Sartelet, J-P Castaigne, R Béliveau

**Affiliations:** 1 Laboratoire de Médecine Moléculaire, Chemistry Department, Université du Québec à Montréal, C.P. 8888, Succ. Centre-Ville, Montréal, QC, Canada H3C 3P8; 2 Angiochem, Inc., Research Department, 201 President-Kennedy Avenue, Suite PK-R220, Montréal, QC, Canada H2X 3Y7; 3 Cerebrovascular Research Group, Institute for Biological Sciences, National Research Council of Canada, 1200 Montreal Road, Ottawa, ON, Canada K1A 0R6; 4 Department of Pathology, CHU Sainte-Justine, Université de Montréal, 3175 Côte Ste-Catherine, Montréal, QC, Canada H3T 1C5

**Keywords:** brain cancer, drug, cancer phenotype, Angiopep-2, blood–brain barrier, low-density lipoprotein receptor-related protein 1 (LRP1)

## Abstract

**Background:**

ANG1005 consists of three molecules of paclitaxel conjugated via ester bonds to the 19-amino-acid peptide Angiopep-2. The new chemical agent has been shown to cross the blood–brain barrier (BBB) by receptor-mediated transcytosis via low-density lipoprotein receptor-related protein 1 (LRP1). The experiments here examined the role of LRP1 in the subsequent endocytosis of drug into cancer cells.

**Methods:**

Localisation of ANG1005 and Angiopep-2 was examined by immunohistochemistry and *in-vivo* near-infrared fluorescence imaging in mice carrying orthotopic glioma tumours. Transport of ANG1005 and Angiopep-2 was examined in U87 glioblastoma cell lines.

**Results:**

Systemically administered ANG1005 and Cy5.5Angiopep-2 localised to orthotopic glioma tumours in mice. The glioma transplants correlated with high expression levels of LRP1. Decreasing LRP1 activity, by RNA silencing or LRP1 competitors, decreased uptake of ANG1005 and Angiopep-2 into U87 glioblastoma cells. Conversely, LRP1 expression and endocytosis rates for ANG1005 and Angiopep-2 increased in U87 cells under conditions that mimicked the microenvironment near aggressive tumours, that is, hypoxic and acidic conditions.

**Conclusion:**

ANG1005 might be a particularly effective chemotherapeutic agent for the wide array of known LRP1-expressing brain and non-brain cancers, in particular those with an aggressive phenotype.

Drug therapy typically has limited efficacy against malignant brain tumours. In part, this is due to the blood–brain barrier (BBB), a unique feature of brain capillary endothelia that prevents many therapeutic agents from crossing brain capillaries into surrounding parenchyma (Bart *et al*, 2000). To circumvent this problem, we have used a novel drug development platform to create new chemical entities (NCEs) with increased brain penetration to treat brain cancers (Demeule *et al*, 2008b). The platform is based on Angiopep-2, a proprietary 19-amino-acid peptide (TFFYGGSRGKRNNFKTEEY) that uses an endocytic receptor in brain capillary endothelial cells, called low-density lipoprotein receptor-related protein 1 (LRP1), to cross the capillary endothelium by receptor-mediated transcytosis (Demeule *et al*, 2008a). The first agent developed in the platform, ANG1005, comprising one Angiopep-2 peptide conjugated via cleavable ester bonds to three molecules of paclitaxel (Regina *et al*, 2008). Two other new agents, ANG1007 and ANG1009, were generated using the same Engineered Peptide Compounds (EPiC) platform and differ from ANG1005 in their active anticancer moieties: doxorubicin and etoposide, respectively (Che *et al*, 2010).

The Angiopep-2 peptide was derived from the Kunitz domain, a known ligand of LRP1 (Warshawsky *et al*, 1994), and has been shown to exhibit transcytosis capacity in an *in-vitro* model of the BBB (Demeule *et al*, 2008a, 2008b) and to cross the BBB *in vivo* by LRP1 receptor-mediated transcytosis in mice (Demeule *et al*, 2008a). A second 19-amino-acid peptide, Angiopep-7, differs from Angiopep-2 in two lysine-to-arginine substitution mutations at positions 10 and 15 and accumulates in brain parenchyma 85% less efficiently (Demeule *et al*, 2008a, 2008b). The observed differences in *in-vivo* transcytosis activities between Angiopep-2 and Angiopep-7 were maintained when the peptides were conjugated to Cy5.5 (1 kDa) (Bertrand *et al*, 2010), a near-infrared fluorescent probe that is unable to cross the BBB on its own (Abulrob *et al*, 2008); in these experiments, Cy5.5Angiopep-2 distributed relatively homogenously throughout cerebral parenchyma after intravenous injection, whereas Cy5.5Angiopep-7 remained primarily associated with cerebral capillaries. Angiopep-7-labelled Cy5.5, therefore, provides a reliable negative control in brain uptake experiments. Angiopep-2 has also been used in other novel approaches for targeting NCEs into brain tissue. For instance, complexing DNA nanoparticles (Ke *et al*, 2009) or micelles (Shen *et al*, 2011) with Angiopep-2 has been shown to stimulate transport across the BBB. Follow-up studies are required, but both of these latter approaches may have the potential to further broaden the utility of the EPiC technology in delivering NCEs to brain tissue for an array of neurological diseases.

LRP1, the receptor for Angiopep-2, is highly expressed *in vivo* in brain endothelial cells, as well as in brain tumour cells, neurons, and astrocytes; it is also expressed in the lung, ovary, uterus, and liver (Acarregui *et al*, 2003; Wilhelmus *et al*, 2007). Low-density lipoprotein receptor-related protein 1, a large receptor of 600 kDa, is a member of the low-density lipoprotein-receptor family and was initially characterised as a clearance receptor for chylomicron remnants (Mahley and Huang, 2007) and *α*2-macroglobulin–proteinase complexes (Strickland *et al*, 1990). Competition studies found that activated *α*2-macroglobulin, a specific ligand for LRP1, and Angiopep-2 share the same receptor (Demeule *et al*, 2008a). However, in *in-vitro* cell culture, most cancer cells express little or no LRP-1 protein, although LRP1 expression may increase greatly in xenografts in severe combined immunodeficient (SCID) mice; LRP1-silenced cancer cells formed tumours that metastasised to the lungs in SCID mice, but the metastases failed to enlarge, suggesting a cell growth or survival deficiency (Montel *et al*, 2007).

The new anticancer Angiopep-2 agent ANG1005 has an antineoplastic potency similar to that of paclitaxel against a variety of human cancer lines (Regina *et al*, 2008). *In vivo* in mice, ANG1005 penetrates into brain parenchyma more rapidly and to a greater extent than unconjugated paclitaxel (Regina *et al*, 2008; Thomas *et al*, 2009). The enhanced penetration of ANG1005 relative to paclitaxel correlates with an increased survival of mice bearing intracerebral implants of U87MG glioblastoma cells or NCI-H460 lung carcinoma cells (Regina *et al*, 2008). Thomas *et al* (2009) recently determined the rate at which ANG1005 transports across the BBB in rats using an *in-situ* brain perfusion methodology. Interestingly, the authors also showed an increased uptake of ANG1005 into tumour tissue of mice bearing brain metastases of the aggressive human breast cancer cell line MDA-MB-231.

This study characterised the involvement of the tumour and the tumour microenvironment in the uptake of Angiopep-2 and ANG1005 in mice brain. For these studies, we chose glioblastoma U87 as a model system, which expresses LRP1 in abundance *in vitro*, and examined the brain biodistribution pattern of ANG1005 and Angiopep-2 bearing human glioma xenografts. As was the case with secondary brain metastases, ANG1005 entered glioma cells after passing through the BBB. Intriguingly, during the course of these studies, we made the novel observation that Angiopep-2 conjugates sequestered at significantly higher levels in this tumour type than in contralateral tumour-free cerebral tissue. Furthermore, *in-vitro* experiments on U87 human glioblastoma cell lines demonstrated that uptake of ANG1005 was stimulated by acidic, hypoxic, and growth factor-starved conditions commonly associated with an aggressive tumour phenotype, whereas the silencing of LRP1 decreased ANG1005 uptake into cancer cells. These data support the idea that ANG1005 might be particularly effective at targeting gliomas and, potentially, other tumour types, particularly those expressing LRP1.

## Materials and methods

### Animals

Male CD-1 nude mice (Charles River Canada, St-Constant, QC, Canada; 6–8-week old) from Charles River Canada were maintained in a pathogen-free environment and handled in accordance with guidelines from the Canadian Council on Animal Care (http://www.ccac.ca/en_/standards/guidelines). Nude mice protocols were approved by the Institutional Animal Care and Use Committee of Université du Québec à Montréal, which is composed of animal care professionals and external observers. The protocols were reviewed every 2 years to ensure proper maintenance and used the 3r principles.

### Labelling of Angiopep-2 and Angiopep-7 with Cy5.5

After solubilisation in dimethylsulphoxide, Angiopep-2 and Angiopep-7 were incubated with inversion with NHS-Cy5.5 at a ratio of 1.2 : 1 in the presence of excess triethylamine (4 h at room temperature in the dark followed by an overnight incubation at 4°C). Coupling was terminated with 4 M hydroxylamine (30 min incubation with inversion). Labelled peptides were subsequently purified by HPLC, lyophilised, and stored at −20°C.

### Iodination of Angiopep-2 and ANG1005

Angiopep-2 in PBS pH 7.4 and ANG1005 in acidified DMSO pH 5.5 were radiolabelled by standard procedures using Iodination Beads from Pierce Chemical (Rockford, IL, USA). In brief, beads were washed twice with 3 ml of PBS on a Whatman filter and resuspended in 60 *μ*l of PBS. Na^125^I (1 mCi) from GE Healthcare (Baie d’Urfé, QC, Canada) was added to the bead suspension for 5 min at room temperature. Iodination was initiated by adding 100 *μ*g of peptide (80–100 *μ*l) diluted in 0.1 M phosphate buffer solution, pH 6.5. After incubation for 10 min at room temperature, beads were removed and the supernatants (^125^I-Angiopep-2) were purified by subsequent hydrophobic chromatography using 30 RPC resin (GE Healthcare) or (^125^I-ANG1005) were purified by repeated (3 × ) water precipitation and centrifugation and suspended in PBS pH 5.5 with 0.1% DMSO to remove free iodine. Quantification and integrity of purified radiolabelled peptides were confirmed by HPLC on a C18 column.

### Intracranial model of U87MG.EGFRvIII glioblastoma in nude mice

For the animal model of high-grade glioma, the U87MG.EGFRvIII cell line, which overexpresses EGFRvIII, was used to assess peptide uptake. Cells were brought into suspension at a final concentration of 1 × 10^4^ cells per *μ*l PBS and were kept on ice until injection. For intracerebral stereotactic implantation of U87MG.EGFRvIII, male CD-1 nude mice were subjected to isofluorane deep anaesthesia, the scalp was swabbed with iodine and alcohol, and the skin was incised. A 10-*μ*l syringe was used to inoculate 5 *μ*l of U87MG.EGFRvIII cell suspension into the corpus striatum in the right hemisphere (3.0 mm deep; 1 mm anterior and 2 mm lateral to the bregma). The skin was sutured with three knots, followed by application of tissue glue and local analgesia. The animals were imaged 10 days after tumour implantation.

### *In-vivo* near-infrared fluorescence imaging of mice bearing U87MG.EGFRvIIII brain tumours

In all, 100 *μ*g of each Cy5.5-labelled peptide was injected via the tail vein in mice bearing 10-day-old U87MG.EGFRvIII brain tumours. *In-vivo* imaging studies were performed using a small animal time-domain eXplore Optix MX2 pre-clinical imager (Advanced Research Technologies, Montreal, QC, Canada) at 0.5, 1, 4, and 24 h after injection. For imaging, mice were first anesthetised with isofluorane and then positioned on an animal stage in a chamber allowing for maintenance of gaseous anaesthesia. In all imaging experiments, a 670-nm pulsed laser diode with a repetition frequency of 80 MHz and a time resolution light pulse of 12 ps was used for excitation. The fluorescence emission at 700 nm was collected by a highly sensitive time-correlated single photon counting system and detected through a fast photomultiplier tube. The data were recorded as temporal point-spread functions and the images were reconstructed as fluorescence concentration maps using ART Optix Optiview analysis software 2.0 (Advanced Research Technologies).

### Brain extraction of ANG1005

ANG1005 was injected into the tail vein of CD-1 or nude mice carrying intracerebral implants of U87 glioblastoma cells. At various times after injection, mice were killed, brains were surgically removed, and brain tissues were dissected and maintained in cold saline containing protease inhibitors until processing. For comparisons of normal and tumour brain tissue, the right hemisphere (implanted hemisphere) was compared with the left hemisphere (non-implanted hemisphere). To extract ANG1005, tissues were mechanically homogenised on ice in homogenisation buffer (0.1 M Tris–HCl, pH 5.0, 50 mM sucrose, 0.003% Tween-80, and protease inhibitor cocktail). Acetonitrile (ACN) 100% was added to a final concentration of 65% (v/v). The mixture was stirred on a nutating mixer for 10 min in a cold room and then centrifuged at 3000 r.p.m. for 5 min. Acetonitrile supernatants were collected and either analysed directly or dried by evaporation in a rotative evaporator (Genevac Inc., Gardiner, NY, USA) and resuspended in DMSO 100%. Subsequent analysis was conducted by HPLC or LC/MS/MS.

ANG1005 was analysed by HPLC on a Zorbax 300SB-CN (50 × 4.6 mm, 5 *μ*m) column (Agilent Technologies, Inc., Mississauga, ON, Canada). Mobile phase A was a mixture of Milli-Q type water and acetonitrile (47.5%/52.5%) containing 7 mM ammonium formate and 0.05% (v/v) TFA. Mobile phase B was a mixture of Milli-Q type water and acetonitrile (10%/90%) containing 7 mM ammonium formate and 0.05% (v/v) TFA. Chromatographic separation was performed using a gradient mode at 70°C at a flow rate of 1.000 ml min^−1^. Detection was made with a tandem mass spectrometry detector API 4000 (AB Sciex, Concord, ON, Canada). The concentration of ANG1005 in the samples was determined using a standard curve (0.50–50 *μ*g ml^−1^) that was confirmed with spiked QC standards.

### Capillary depletion

Isolated brain tissue was homogenised on ice in Ringer's N-2-hydroxyethylpiperazine-N′-2-ethanesulphonic acid (HEPES) buffer with 0.1% BSA in a glass homogenizer. Brain homogenate was mixed thoroughly with 35% Dextran 70 (50 : 50) and centrifuged at 5400 *g* for 10 min at 4°C. The supernatant (composed of brain parenchyma) and the pellet (representing capillaries) were carefully collected for further analysis.

### Immunohistochemistry

Brain sections were processed for immunohistochemistry 24 h after intravenous bolus injection of Cy5.5Angiopep-2 (100 *μ*g) as described (Tarasenko *et al*, 2008). Avidin–biotin non-specific binding was prevented using a blocking kit according to the manufacturer's protocol (Vector Laboratories, Inc., Burlingame, CA, USA). Sections were incubated at 4°C overnight with a primary rabbit antibody against Ki-67 (1 : 300) or Angiopep-2 (1 : 500). Before use, the polyclonal anti-Angiopep-2 antibody had been purified using Angiopep-2-coupled Sepharose beads to remove non-specific binding activity. The secondary antibody was a biotin-conjugated goat anti-rabbit IgG. After treatment with antibodies, slides were stained with the ABC peroxidase system and developed with diaminobenzidine (Vector Laboratories, Inc.).

### Peptide detection by immunofluorescence

Twenty-four hours after intravenous bolus injection of Cy5.5Angiopep-2 (100 *μ*g), animals were perfused for 10 min with heparinised saline and 10% formalin. Vibratome brain sections were obtained (50 *μ*m thickness) and viewed in the near-infrared mode (a 660- to 680-nm excitation filter and a 700-nm longpass emission filter) using Olympus 1X81 an inverted motorized microscope (Olympus Canada, Inc., Richmond Hill, ON, Canada) and analysed using ImagePro 6.2 (Media Cybernetics, Inc., Bethesda, MD, USA). DAPI (1 mg ml^−1^) was used to stain the DNA nuclei.

### Immunoblotting procedures

Proteins from treated cells were separated by sodium dodecyl sulphate–polyacrylamide gel electrophoresis. After electrophoresis, proteins were electrotransferred onto polyvinylidene difluoride membranes, which were then blocked for 1 h at 20°C with 5% non-fat dry milk in Tris-buffered saline (150 mM NaCl, 20 mM Tris–HCl, pH 7.5) containing 0.3% Tween-20 (TBST). Membranes were further washed in TBST and incubated with the primary antibodies (2.5 *μ*g ml^−1^). The LRP1 monoclonal antibody (mAb) was purchased from Research Diagnostics (Flanders, NJ, USA), in TBST containing 3% BSA and 0.02% NaN_3_, followed by a 1-h incubation with horseradish peroxidase-conjugated anti-rabbit IgG (1 : 2500 dilution) in TBST containing 5% non-fat dry milk. Three washes with TBST were performed after the incubations with the primary and secondary antibodies. Immunoreactive material was visualised by enhanced chemiluminescence (GE Healthcare).

### LRP1 silencing

*In-vitro* silencing of LRP1 was carried out by standard methodologies (Song *et al*, 2009). The sequences of oligonucleotides were GCAGUUUGCCUGCAGAGAUtt (sense) and AUCUCUGCAGGCAAACUGCtt (antisense). Human U87 glioblastoma cells were grown to 50% confluence and were transfected with LRP1 silencing RNA (siRNA) using Lipofectamine 2000 (Life Technologies Corporation, Carlsbad, CA, USA) according to the manufacturer's specifications. After 48 h of transfection, cells were used for subsequent assays. Expression of proteins was assessed by western blot analysis.

### Cell culture

The human U87 glioblastoma, Hepg2, and MG63 cell lines were purchased from American Type Culture Collection, and cultured under a humidified atmosphere of 5% CO_2_ in modified Eagle's medium containing 10% FBS, 2 mM glutamine, 100 U ml^−1^ penicillin, and 100 mg ml^−1^ streptomycin.

#### pH variation

Cells were incubated in medium in which the sodium bicarbonate had been replaced with Ringer/HEPES buffer (150 mM NaCl, 5.2 mM KCl, 2.2. mM CaCl_2_, 0.2 mM MgCl_2_·6H_2_O, 6 mM NaHCO_3_, 2.8 mM glucose, and 5 mM HEPES). The pH of the incubation medium used in the uptake experiments was varied from 8.0 to 6.75 with 1 N hydrochloric acid or NaOH.

#### Hypoxic conditions

Subconfluent cells were incubated for 24 h in an anaerobic box with oxygen maintained at 1% by a compact gas oxygen controller Proox model 110 (Reming Bioinstruments Co., Redfield, NY, USA) and a residual gas mixture composed of 94% N_2_ and 5% CO_2_.

#### Serum deprivation

Subconfluent cells were grown overnight in medium lacking serum.

### [^125^I]-Angiopep-2 or [^125^I]-ANG1005 uptake assay

Cells were seeded in a 24-well plate (5 × 10^4^ cells per well) in appropriate media and fed for 3 days. To test for Angiopep-2 uptake, the medium was aspirated off and replaced with Ringer/HEPES buffer adjusted to pH 7.4 with NaOH. Subconfluent cells were incubated at 37°C with [^125^I]-Angiopep-2 (500 nM) or [^125^I]-ANG1005 (500 nM, in 0.1% DMSO) in the presence or absence of unlabelled Angiopep-2 at various concentrations (quadruplicate determinations) for a 2- to 5-min period. Cells were then lysed in 0.3 M NaOH and shaken with a Titer Plate Shaker (Lab-Line Instruments, Inc., Melrose Park, IL, USA) for 30 min at room temperature. Uptake assays in cells grown under differing pH conditions were carried out after three washes. Uptake assays in cells grown under anaerobic conditions were performed on cells that had been incubated under normal oxygen conditions for 2 h to allow the cells time to recuperate. Competitive inhibition of [^125^I]-Angiopep-2 uptake was performed with receptor-associated protein (RAP) (40 *μ*g ml^−1^ or 1 *μ*M) with a prewash of 1 h in the Ringer/HEPES buffer to clean surface receptor of the cells. Results were quantified with a Wizard 1470 Gamma counter (Perkin-Elmer, Woodbridge, ON, Canada). Background radiation of unlabelled cells was subtracted from [^125^I]-Angiopep-2 values (quadruplicate determinations).

### cDNA synthesis and real-time quantitative RT–PCR

For cDNA synthesis, 1 *μ*g total extracted RNA was reverse transcribed into cDNA using a high capacity cDNA reverse transcription kit (Applied Biosystems, Foster City, CA, USA). cDNA was stored at −80°C for PCR. Gene expression was quantified by real-time quantitative PCR using iQ SYBR Green Supermix (BIO-RAD, Hercules, CA, USA). DNA amplification was carried out using an Icycler iQ5 (BIO-RAD) and product detection was performed by measuring the binding of the fluorescent dye SYBR Green I to double-stranded DNA. All the primer sets were provided by QIAGEN (Toronto, ON, Canada). The relative quantities of target gene mRNA relative to an internal control, 18S ribosomal RNA, were measured by following a *C*_T_ method. An amplification plot (fluorescence signal *vs* cycle number) was drawn. The difference (*C*_T_) between the mean values in the triplicate samples of target gene and those of 18S ribosomal RNA was calculated by iQ5 Optical System Software version 2.0 (BIO-RAD), and the relative quantified value (RQV) was expressed as 2^−Δ*C*_T_^.

### Data analysis

Data are expressed as mean±s.e.m. Statistical analyses were performed using Student's *t*-test when one group was compared with the control group. To compare two or more groups with the control group, one-way analysis of variance with Dunnett's *post hoc* test was used. In addition, curve slopes were used to determine whether two curves were statistically different. All statistical analyses were performed using GraphPad Prism version 4.0C for Macintosh (GraphPad Software Inc., San Diego, CA, USA). Significance was assumed for *P*-values <0.05.

## Results

### Brain localisation of Cy5.5Angiopep-2 in mice with orthotopic human glioma tumours

**Figure 1:**
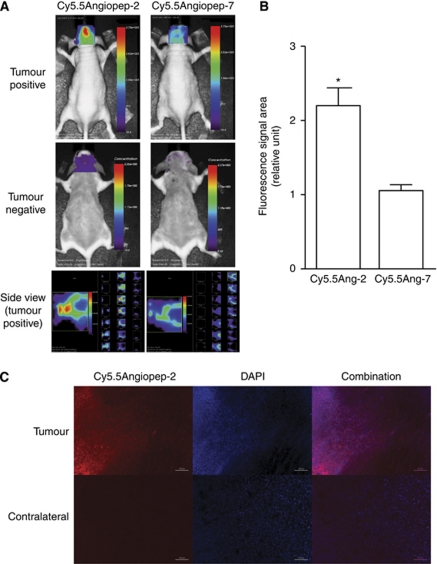
Localisation of Cy5.5Angiopep-2 and Cy5.5Angiopep-7 in mice carrying U87 human glioma tumour xenografts and in control mice. (**A**) Non-invasive time-domain fluorescence images (dorsal aspect) of mice 24 h after intravenous injection of Cy5.5Angiopep-2 (100 *μ*g; left column) or Cy5.5Angiopep-7 (100 *μ*g; right column) in tumour-positive (top row) or tumour-negative (middle row) animals. The images show peak fluorescence in relative units. The bottom row contains virtual optical tomography lateral images (*Z*-axis sections) of tumour-bearing mice 24 h after intravenous injection of Cy5.5Angiopep-2 (100 *μ*g; left panel) and Cy5.5Angiopep-7 (100 *μ*g; right panel). The series of images on the right represent 0.5 *μ*m sections (transverse, top-to-bottom). (**B**) Semiquantitative analysis of Cy5.5Angiopep-2 and Cy5.5Angiopep-7 brain localisation in tumour-positive animals analysed by non-invasive time-domain fluorescence imaging. Results are based on the area of intensity of relative fluorescence in the brain. Data are expressed as the mean±s.e.m. of three experiments (^*^P<0.05). (**C**) Fluorescence microscopy of brain sections 24 h after intravenous bolus injection of Cy5.5Angiopep-2 (100 *μ*g). (Cy5.5Angiopep-2, top row) Brain tumour section subjected to fluorescence microscopy. Cy5.5Angiopep-2 is detected as red spots. (DAPI, top row) The same tumour section stained with DAPI and subjected to fluorescence microscopy. Cell nuclei are labelled in blue. (Combination, top row) Superimposition of the Cy5.5Angiopep-2 and DAPI images to the left. (Bottom row) The same set of experiments as described above, but on a section from the contralateral hemisphere.

The *in-vivo* biodistribution patterns of Cy5.5Angiopep-2 and Cy5.5Angiopep-7 were analysed in mice after a single systemic injection of either agent using *in-vivo* optical imaging (Figure 1A, top and middle rows). Twenty-four hours after injection, the Cy5.5Angiopep-2 fluorescence signal in the head region was higher in animals bearing orthotopic glioblastoma xenografts than in control animals lacking tumours, demonstrating a dramatic tumour-specific homing of Angiopep-2 to the brain area. Moreover, this head-specific homing of Cy5.5Angiopep-2 was significantly greater than that observed after injection of the negative control Cy5.5Angiopep-7 (Figure 1B). Nonetheless, a low-level fluorescence signal was observed in the tumour-positive animals 24 h after Cy5.5Angiopep-7 injections (Figure 1A, top row), which appeared to be of higher intensity than the signal observed in tumour-negative animals after injection of either agent (Figure 1A, middle row). It should also be noted that the *in-vivo* assay was sensitive enough to distinguish between apparent brain uptake of Cy5.5Angiopep-2 and Cy5.5Angiopep-7 in tumour-negative animals, as the fluorescence observed for Cy5.5Angiopep-7 was lower than Cy5.5Angiopep-2 and similar to that observed in animals before injection (data not shown).

To further define the region of localisation of Cy5.5Angiopep-2, virtual optical tomography was performed on the mice using lateral optical slices (Figure 1A, bottom row). These studies confirmed that the signal strength for Cy5.5Angiopep-2 was highest in a region overlapping the tumour implant. Furthermore, the highest fluorescence occurred at an intracranial depth (5–6 mm) that corresponded closely with the intracerebral stereotactic implantation of U87 cells.

Immediately after imaging, animals were killed, brains were surgically removed and treated with DAPI to stain for nuclei, and brain slices were analysed by immunofluorescence. Tumour tissue was identified based on increased density of nuclei and hemispheric location, while Cy5.5Angiopep-2 was localised using the far-red fluorescence pattern of the Cy5.5 moiety. Cy5.5Angiopep-2 localised at higher levels within tumour tissue than contralateral normal cerebral tissue (Figure 1C), consistent with a pronounced sequestration of Angiopep-2 into U87 brain tumours. In contrast, 24 h after injection, no residual Cy5.5Angiopep-7 fluorescence was observed in either tumour tissue or contralateral normal cerebral tissue.

### Uptake of ANG1005 in mice with orthotopic human glioma tumours

**Figure 2:**
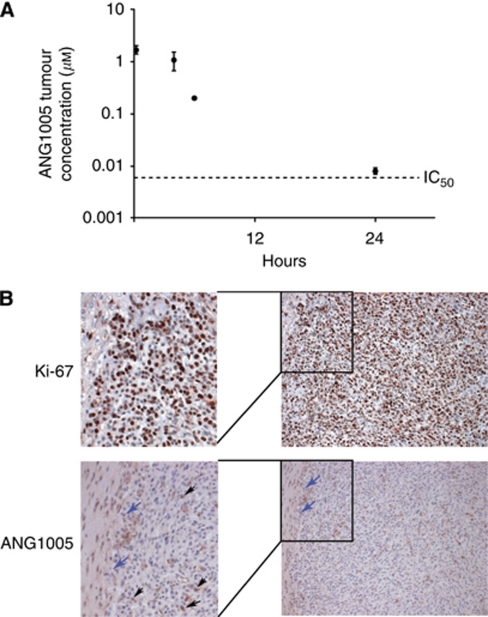
ANG1005 brain uptake and LRP1 expression in mice carrying U87 human glioma tumour xenografts. (**A**) Brain uptake of [^125^I]-ANG1005 measured 0.25, 4, 6, and 24 h after bolus injection. Quantification was by LC/MS/MS. Dash line represent IC_50_ value for ANG1005 with U87 cells *in vitro*. Mean values±s.e.m. are for *n*=3 animals. (**B**) Immunohistochemistry of an implanted glioma using primary antibodies directed against either proliferation marker Ki-67 or Angiopep-2. Analysis was carried out 24 h after intravenous bolus injection of ANG1005 (100 *μ*g). The micrographs in the left column are two-fold digital magnifications of the designated areas of the micrographs in the right column. Blue arrows mark the tumour boundary. Black arrows mark round tumour cells positive for ANG1005.

To confirm that the increased brain localisation was not unique to Cy5.5Angiopep-2, similar experiments were conducted using ANG1005, which contains Angiopep-2 conjugated to three molecules of paclitaxel (Regina *et al*, 2008). In these experiments, animals bearing orthotopic glioma tumours received a single intravenous injection of ANG1005, and the concentration of the drug in brain tissue was assessed by HPLC (Materials and methods) 0.25, 4, 6, and 24 h later (Figure 2A). At 0.25 h, ANG1005 was present in brain tissue at a concentration of 1.67±0.50 *μ*M, demonstrating rapid uptake of the drug. The concentration dropped relatively steadily from that point, reaching a concentration of 0.008±0.002 *μ*M 24 h post-injection. The 0.008-*μ*M concentration at 24 h remained significantly above the known *in vitro* IC_50_ for the U87 human glioblastoma cell line (0.0051 *μ*M) (Regina *et al*, 2008).

Brain slices were also prepared from animals 24 h after injection of ANG1005, and localisation was conducted by immunohistochemistry using a primary antibody against Angiopep-2, which also recognises ANG1005 (Figure 2B). Cancer cells in proliferation were localised using antibody against the cellular proliferation protein Ki-67. Signal from the ANG1005 was observed homogeneously throughout the tumour and stroma and overlapped with the Ki-67 signal, consistent with localisation of ANG1005 to the tumour cells.

### Mechanism of Angiopep-2 transport into U87 human glioblastoma cells

**Figure 3:**
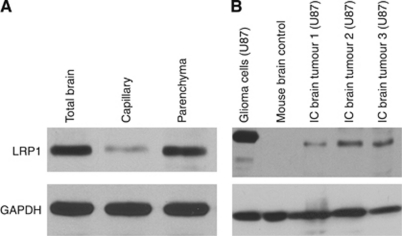
(**A**) Western blot analysis of LRP1 expression in total brain, capillaries, and parenchyma in normal mice. Fractionation of brain tissue was by differential centrifugation through 35% Dextran 70 as described in Materials and methods. Each lane contained 50 *μ*g of protein. (**B**) Western blot analysis of LRP1 in total brain tissue from three animals with intracranial (IC) gliomas, in whole-brain extract from a normal animal, and in proteins extracted from an *in-vitro* culture of U87 cells. Each lane contained 10 *μ*g of protein. Loading control in (**A**) and (**B**) was glyceraldehyde 3-phosphate dehydrogenase (GAPDH).

One potential hypothesis to account for the observed sequestration into orthotopic gliomas proposes that Angiopep-2 conjugates, after penetrating the BBB, interact again with tumour-bound LRP1, which is known to be prevalent in glial lineages (Lillis *et al*, 2005), and subsequently enter the individual cells by endocytosis. To examine this model, we started by quantifying the expression levels of LRP1 protein in animals bearing glioma tumours by western blot analysis. However, since capillary extraction was found to pose technical challenges in animals bearing glioma tumours, brain tissue from normal animals was assessed instead. Thus, when total brain homogenates from normal brains were subjected to differential separation of the brain capillaries (pellet) from the parenchyma (supernatant) (Demeule *et al*, 2008b), the large majority of signal was found in the parenchymal fraction (Figure 3A). Consistent with its relatively high expression in glioma cells (Lillis *et al*, 2005), LRP1 protein levels in total brain tissue were significantly higher in mice carrying glioma tumours than normal animals (Figure 3B), demonstrating that the presence of glioma transplants correlated with increased expression of LRP1.

**Figure 4:**
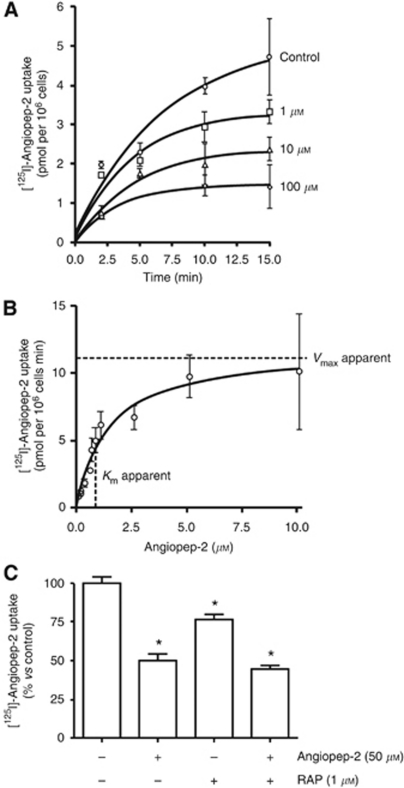
Angiopep-2 uptake into U87 human glioma cells. (**A**) Uptake of [^125^I]-Angiopep-2 (500 nM) measured for 2, 5, 10, or 15 min in the presence of 0 (control), 1, 10, and 100 *μ*M unlabelled Angiopep-2. (**B**) Uptake of [^125^I]-Angiopep-2 (500 nM) measured at 2 min as a function of unlabelled concentration of Angiopep-2. All values were corrected by subtracting the non-specific uptake measured in the presence of unlabelled Angiopep-2 (100 *μ*M). (**C**) Uptake of [^125^I]-Angiopep-2 (500 nM) by itself and in the presence of unlabelled Angiopep-2 (50 *μ*M), preincubated unlabelled RAP (1 *μ*M), and both unlabelled Angiopep-2 (50 *μ*M) and unlabelled RAP (1 *μ*M). Uptake was quantified and expressed as percentage of control. Data are expressed as the mean±s.e.m. of quadruplicate experiments (^*^P<0.05).

Prior studies showed that ANG1005 has a similar cytotoxic effect *in vitro* in U87 human glioblastoma cells as unconjugated paclitaxel (Regina *et al*, 2008), but those studies did not directly examine whether ANG1005 and Angiopep-2 entered cells by an LRP1-mediated mechanism. Here, using for the first time an LRP1 model that expresses high levels of LRP1, we characterised the uptake of [^125^I]-Angiopep-2 into U87 human glioblastoma cells as a function of time in the presence of 0, 1, 10, or 100 *μ*M unlabelled Angiopep-2. The addition of unlabelled Angiopep-2 inhibited the uptake of [^125^I]-Angiopep-2 in a dose-dependent manner, consistent with a saturable receptor-mediated process (Figure 4A). The initial uptake measured at 2 min was plotted as a function of unlabelled Angiopep-2 concentration, resulting in apparent *K*_m_ and *V*_max_ values of 1.2 *μ*M and 12.3 pmol per 10^6^ cells per minute, respectively (Figure 4B).

Uptake of [^125^I]-Angiopep-2 (500 nM) was inhibited by unlabelled Angiopep-2 (50 *μ*M) and by RAP (1 *μ*M), a known LRP1 ligand that competitively inhibits uptake of Angiopep-2 in brain capillary cells (Demeule *et al*, 2008a; Figure 4C). When both competitors were tested together, the inhibition of [^125^I]-Angiopep-2 uptake was similar to that observed with Angiopep-2 alone (Figure 4C), consistent with receptor saturation occurring at an unlabelled Angiopep-2 concentration of ⩽50 *μ*M. The absence of further inhibition in the presence of both inhibitors confirmed prior studies showing that Angiopep-2 and RAP share the same endocytic receptor, that is, LRP1.

**Figure 5:**
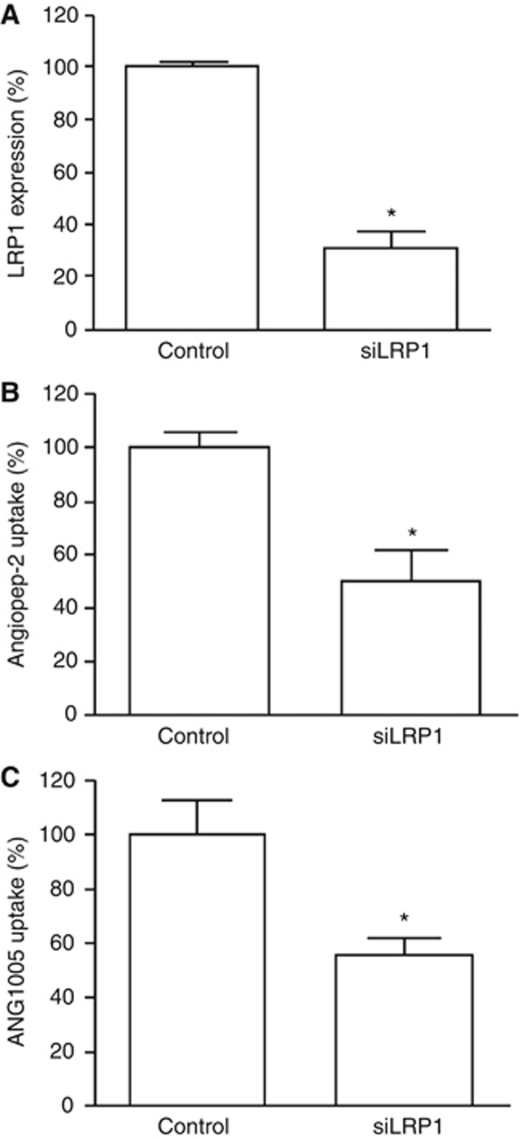
Role of LRP1 in the uptake of Angiopep-2 and ANG1005 into U87 human glioma cells. (**A**) Cells grown for 48 h in the presence or absence of an LRP1 siRNA were quantified for LRP1 expression by western blot. Cells in (**A**) were incubated for 5 min with [^125^I]-Angiopep-2 (500 nM) (**B**) or [^125^I]-ANG1005 (500 nM) (**C**) and uptake was quantified and expressed as percentage of control. Data are expressed as mean±s.e.m. of quadruplicate experiments (^*^P<0.05).

To provide more confirmatory evidence, a parallel approach based on siRNAs was undertaken. Consistent with the prior inhibition studies, western blot analysis indicated that cells containing an LRP1 siRNA, which reduced LRP1 protein levels by ∼70% (Figure 5A), reduced the uptake of both Angiopep-2 (Figure 5B) and ANG1005 (Figure 5C) by ∼50% (*P*<0.05). In combination, the inhibition and siRNA studies provide strong evidence that LRP1-mediated endocytosis is a primary mechanism by which Angiopep-2 and ANG1005 enter glioblastoma cells.

### LRP1 expression and Angiopep-2 conjugate endocytosis in conditions associated with cancer microenvironment

**Figure 6:**
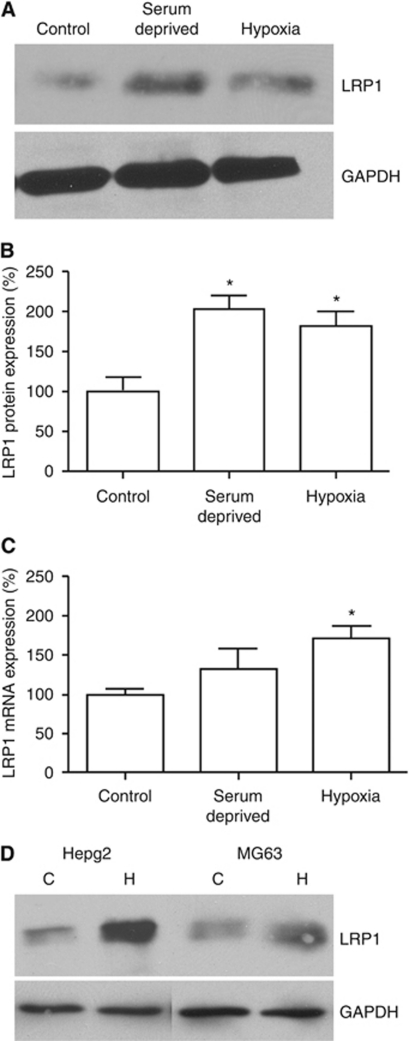
Effect of serum deprivation and hypoxia on LRP1 protein and RNA expression. (**A**) Western blot analysis of LRP1 expression in U87 human glioma cells grown under normal, serum-deprived, and hypoxic conditions. (**B**) LRP1 protein expression was quantified, normalised to GAPDH expression, and expressed as percentage of control. (**C**) LRP1 mRNA expression in U87 human glioma cells grown under normal, serum-deprived, and hypoxic conditions was quantified by real-time quantitative PCR and expressed as percentage of control. (**D**) LRP1 expression in hepatocarcinoma (Hepg2) and osteosarcoma (Mg63) cells under hypoxic (H) and control (C) conditions. Data in (**A**) and (**B**) show the best representatives of at least quadruplicate experiments. Data in (**B**) and (**C**) represent the mean values±s.e.m. of at least quadruplicate experiments (*P<0.05). GAPDH, glyceraldehyde 3-phosphate dehydrogenase-positive loading control.

Gliomas are particularly aggressive tumours, and it was therefore of interest to examine how expression of LRP1 and the transport of Angiopep-2 conjugates were affected by conditions associated with cancer microenvironment, such as hypoxia and low pH. Similarly, many cancer cells are characterised by an ability to grow in low levels of growth hormone, so it was of interest to examine LRP1 expression in cells grown under general serum-deprived conditions, although not strictly an exact replica of *in-vivo* conditions near a tumour. In human U87 glioblastoma cell lines, hypoxia and serum deprivation were associated with statistically significant two-fold increases in LRP1 protein expression (Figures 6A and B). Similarly, at the mRNA level, LRP1 expression increased significantly in hypoxic conditions (62% *P*<0.05) (Figure 6C). The LRP1 mRNA levels increased in serum-deprived conditions as well, but the difference with control did not reach statistical significance (Figure 6C). Expression of LRP1 was assessed in two other cells lines, Hepg2 (hepatocarcinoma) and MG63 (osteosarcoma), to examine whether this phenomenon is generalised in other cancer cells. Under hypoxic conditions, both cancer cell lines increased LRP1 expression by about 110% and 70%, respectively (Figure 6D).

**Figure 7:**
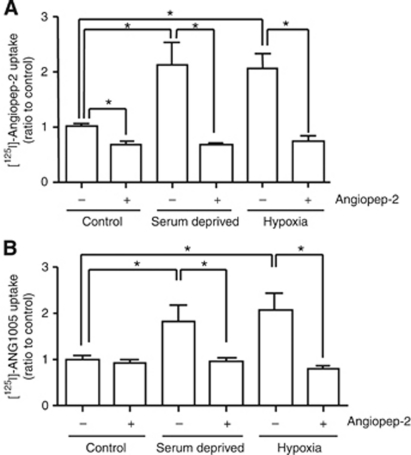
Effect of serum deprivation and hypoxia on Angiopep-2 and ANG1005 uptake. Uptake of [^125^I]-Angiopep-2 (500 nM) (**A**) and [^125^I]-ANG1005 (500 nM) (**B**) into U87 human glioma cells under normal (control), serum-deprived, and hypoxic conditions with (+) or without (−) excess cold Angiopep-2 (50 *μ*M). Data represent the mean values±s.e.m. obtained from three experiments, each run in quadruplicate (*P<0.05).

To further explore the implications of this increased LRP1 expression on endocytosis of Angiopep-2 and ANG1005, we examined human U87 glioblastoma cells and found that the changes in mRNA and protein expression were associated with ∼100% increased uptake of Angiopep-2 (Figure 7A) and ANG1005 (Figure 7B).

**Figure 8:**
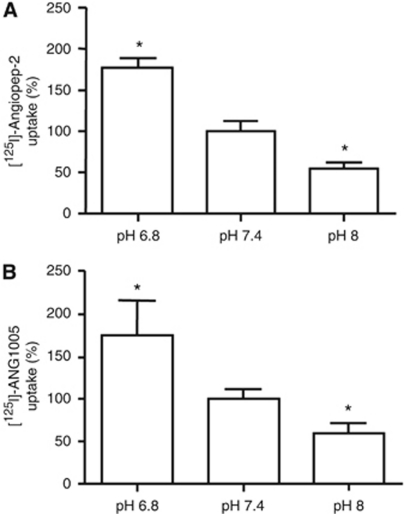
Effect of media pH on Angiopep-2 and ANG1005 uptake. Uptake of [^125^I]-Angiopep-2 (500 nM) (**A**) and [^125^I]-ANG1005 (500 nM) (**B**) into U87 human glioma cells grown in media at pH 6.8, 7.4, and 8.0. Data represent the mean values±s.e.m. as compared to pH 7.4 condition obtained from three experiments, each run in quadruplicate (*P<0.05).

Finally, uptake increased linearly as pH in the surrounding incubation medium decreased from 8.0 to 6.8 for both Angiopep-2 (Figure 8A) and ANG1005 (Figure 8B). Overall, accumulation of Angiopep-2 and ANG1005 was 56% and 62% greater, respectively, at pH 6.8 compared with normal physiological pH (pH 7.4). In the studies examining the effects of pH changes, as well as the prior ones examining serum deprivation and hypoxia, accumulation of Angiopep-2 and ANG1005 was abrogated in the presence of 100-fold excess of unlabelled Angiopep-2 competitor.

## Discussion

Using a combination of *in-vivo* optical imaging and immunolocalisation methodologies, we showed that systemically administered Cy5.5Angiopep-2 and ANG1005 passed through the BBB in mice and subsequently accumulated at higher levels in orthotopic human glioma tumours than in contralateral non-tumour brain tissue. Several lines of evidence indicate that this sequestration involved LRP1-mediated endocytosis of the Angiopep-2 conjugates into individual tumour cells. First, orthotopic glioma tumours exhibited increased uptake of Angiopep-2 and ANG1005 compared with control. Second, LRP1 protein levels were dramatically higher in brain tissue from mice carrying orthotopic glioma tumours than normal mice. Third, ANG1005 was transported into U87 human glioblastoma cells via a saturable receptor-mediated process that was inhibited by adding RAP, a known LRP1 ligand that competitively inhibits uptake of Angiopep-2 in brain capillary cells (Demeule *et al*, 2008a), or by reducing LRP1 expression using siRNA. Finally, changes in microenvironmental conditions near tumour cells that increased LRP1 expression simultaneously increased uptake of Angiopep-2 and ANG1005.

The uptake of ANG1005 and Cy5.5Angiopep-2 into tumour tissue was dramatically improved compared with normal tissue. Both optical imaging studies on Cy5.5Angiopep-2 in live animals and direct biochemical analyses of ANG1005 concentrations subsequent to intravenous administration indicated fast penetration into general brain tissue. Moreover, the high fluorescence signal detected at 24 h after Cy5.5Angiopep-2 injection was consistent with a relatively rapid accumulation in tumour tissue followed by retention of the peptide (Regina *et al*, 2008). The preceding observations confirm previous data from *in-vitro* assays and *in-situ* brain perfusion studies, which demonstrated that ANG1005 exhibited rapid transport kinetics across the BBB (Demeule *et al*, 2008a, 2008b; Thomas *et al*, 2009) and that the drug was able to transport into tumour tissue in mice bearing metastases of human breast cancer cells (Thomas *et al*, 2009). Evidence from this study also corroborates earlier studies in our laboratory (Bertrand *et al*, 2010), indicating that the entire Cy5.5Angiopep-2 molecule, and not a breakdown Cy5.5 product, is the transport substrate. The fluorescence lifetimes (*τ*) of Cy5.5 and Cy5.5Angiopep-2 are sufficiently different (1.1 and 1.5 ns, respectively) to be able to differentiate between free dye and dye-peptide conjugate. In the studies described here, the measured *τ* in the brain appeared to originate exclusively from Cy5.5Angiopep-2 (data not shown). The retention of ANG1005 in brain tissue was also noteworthy. Specifically, the concentration of ANG1005 in total hemispheric brain tissue 24 h after a single systemic injection (0.008±0.002 *μ*M) remained above the known *in vitro* IC_50_ for the U87 human glioblastoma cell line (0.0051 *μ*M) (Regina *et al*, 2008). Owing to the tumour-specific localisation of drug, the 0.008-*μ*M value likely underestimated the concentration of ANG1005 within the glioma itself.

Many brain tumours contain capillaries with a compromised BBB, which is the underlying cause of vasogenic oedema (Laterra and Goldstein, 2000; Provenzale *et al*, 2005). It might, therefore, be argued that the enhanced uptake of Cy5.5Angiopep-2 merely reflected the localised degradation of the BBB in the vicinity of the xenograft tumour tissue. However, this conclusion is inconsistent with our data on the negative control molecule, Cy5.5Angiopep-7. The latter peptide, which differs from Cy5.5Angiopep-2 by only two amino-acid substitutions (Demeule *et al*, 2008b), exhibited significantly lower penetration of tumour tissue and overall brain parenchyma in animals carrying orthotopic glioma tumours. Thus, a disrupted BBB is not enough to account for the full range of brain tumour localisation we observed with Angiopep-2 conjugates, but instead required specific targeting and/or retention mediated by Angiopep-2. In the specific case of malignant gliomas, which are commonly sensitive to chemotherapeutics *in vitro* but not *in vivo* under clinical conditions, spread of cancer cells almost always occurs locally along neuronal fibres within the brain, with metastatic lesions rarely forming outside the CNS (Tange *et al*, 2009). Moreover, it should be noted that even a compromised BBB remains a very formidable diffusion barrier (Palmieri *et al*, 2006). Assuming a 10-fold elevation in permeability, which is in line with published values for capillaries within brain tumours (Palmieri *et al*, 2006), the disordered BBB still exhibits a permeability to polar solutes that is 10^2^–10^5^ times less than that of peripheral capillaries (Palmieri *et al*, 2006).

This remaining permeability barrier may be due, in part, to the continued expression of active efflux pumps in the capillary endothelium, as demonstrated in a number of brain tumours. Additionally, interstitial fluid pressures can be >50 mmHg in peritumoural areas compared with 2 mmHg in normal brain tissue (Muldoon *et al*, 2007), a pressure differential that has been shown to significantly reduce diffusion of drugs into tumour tissue (Ali *et al*, 2006; Navalitloha *et al*, 2006). None of these latter phenomena, however, prevented ANG1005 from colocalising with tumour cells, again supporting a role for active uptake of the Angiopep-2 conjugate. Importantly, the homogeneous distribution of ANG1005 throughout the tumour mass also argues that an active transport process, such as one mediated by LRP1, participates in the transport under the unique conditions present in this aggressive tumour type.

The LRP1 is a multifunctional receptor that is expressed in a wide range of tissues, as well as in multiple malignancies, including colon cancer, renal cancer, and melanocytic tumours (de Vries *et al*, 1996; Desrosiers *et al*, 2006; Obermeyer *et al*, 2007). This broad distribution pattern in various cancers is consistent with increasing evidence that implicates LRP1 as a central player in oncogenesis, tumour progression, and metastasis (Herz and Strickland, 2001). Mechanistically, a major function of LRP1 appears to lie in the removal of proteinase and proteinase inhibitor complexes, including matrix metalloproteinases and urokinase-type plasminogen activator, raising the possibility that the molecule acts as a multifunctional scavenger receptor (Herz and Strickland, 2001; Song *et al*, 2009). Because of the importance of extracellular proteases in tumour progression and metastasis, this activity may account for the critical role of LRP1 in aggressive tumours (Duffy *et al*, 2000). Additionally, LRP1 has been shown to interact with scaffolding and signalling proteins, thereby functioning as a co-receptor with other cell surface or integral membrane proteins involved in growth control and invasion (Lillis *et al*, 2005). Although the precise mechanisms for LRP1 in malignancy remain controversial, accumulating evidence of its role in cancer progression encourage further studies on the interplay between an aggressive phenotype and uptake of ANG1005 and other EPiC agents.

The enhanced transport capacity observed in this study was likely attributable to the presence of high LRP1 levels in individual glioblastoma cells. With apparent *K*_m_ and *V*_max_ values of 1.2 *μ*M and 12.3 pmol per 10^6^ cells per minute, respectively, the transport capacity of U87 cells was 10-fold higher than brain capillary endothelial cells (Bertrand *et al*, 2010). Furthermore, the *V*_max_ was obtained rapidly. These results are consistent with a relatively rapid accumulation in tumour tissue followed by retention of the peptide (Regina *et al*, 2008). Moreover, *in-vitro* inhibition of uptake with both LRP1 competitor and LRP1 silencing indicates that uptake occurs via the same receptor. Taken together, the data as a whole argue strongly that LRP1-mediated endocytosis is a primary mechanism by which Angiopep-2 and ANG1005 enter glioblastoma cells. The impact of systemically administered Angiopep-2 conjugates on LRP1-mediated transport of endogenous proteins remains unknown, although the potential to act as general competitive inhibitors may be low. Most LRP1 receptors are found in rapidly recycling vesicles, which move to the cell surface in response to a signal, bind to one of many ligands, participate in endocytosis, and finally recycle back to the beginning of the cycle (Bilodeau *et al*, 2010). This rapid turnover mechanism is likely difficult to inhibit with exogenous ligands in a clinically significant manner, but this prediction requires further study.

Rapidly growing cancer cells can create microenvironmental conditions that differ from normal tissues. Thus, the microenvironment of a tumour is generally more hypoxic (Mekhail *et al*, 2004; Kapitsinou and Haase, 2008; Vaupel, 2008) and acidic than normal tissues. Moreover, cells within tumours generally have less access to exogenous growth factors and nutrients (Carpenter and Cantley, 2005). These conditions generate selective pressures that favour tumour cell variants with aggressive phenotypes, that is, cells with more robust growth, increased migration capacity, and greater invasiveness (Gupta and Massague, 2006). To address whether this microenvironmental conditioning had a role in our observations, we examined how LRP1 expression and ANG1005 endocytosis in glioblastoma cells were affected by conditions known to be present in aggressive tumours. We found that glioblastoma cell lines exposed to hypoxic or serum-deprived conditions upregulated LRP1 expression and exhibited significantly increased uptake of Angiopep-2. In two other cancer cell lines (bone and liver), we found that hypoxia similarly upregulated expression of LRP1. In a prior study, LRP1 expression was shown to be dramatically increased in mouse xenografts relative to the same cells grown in standard *in-vitro* culture conditions (Montel *et al*, 2007). The latter induction of LRP1 was a response to the hypoxic conditions in the fast-growing tumours and could be recapitulated *in vitro* under hypoxic conditions (Montel *et al*, 2007). Furthermore, hypoxia and HIF-1*α* overaccumulation was also shown to induce LRP1 promoter activity in other types of cells (Castellano *et al*, 2011). These studies, combined with the fact that LRP1 appears to be encoded by a general hypoxia-regulated gene (Koong *et al*, 2000; Wykoff *et al*, 2000; Zatyka *et al*, 2002), suggest that the microenvironmental conditions surrounding aggressive gliomas and other tumours might stimulate uptake of Angiopep-2 and ANG1005. Thus, LRP1 may be an especially attractive target for selective receptor-mediated drug delivery to tumours because of the specific microenvironment created by the tumour itself.

Its broad tissue distribution also means that LRP1 is present in many non-cancerous tissues, as well. Consequently, the therapeutic window for ANG1005 will reflect a balance between specific targeting to tumour cells *vs* ‘non-specific’ targeting to healthy tissue. However, the potentially enhanced transport of the drug under hypoxic conditions may mean that uptake into rapidly growing cancer cells will be favoured. It will be of significant interest in the future to determine whether the tumour-specific homing identified in this study translates into a more favourable safety profile for the drug. Ultimately, answering this question will require completion of large-scale controlled clinical trials. It is of interest to note, in this regard, that ANG1005 has been evaluated in two separate phase 1 multi-center, open-label, dose-escalation trials to identify the maximum tolerated dose and to obtain data on safety, tolerability, and preliminary efficacy in patients with either heavily pretreated advanced solid tumours with brain metastases or recurrent malignant glioma (Drappatz *et al*, 2010; Sarantopoulos *et al*, 2010). Toxicities related to ANG1005 were similar to other taxanes, such as paclitaxel, with dose-limiting toxicity due to neutropenia.

In conclusion, we have characterised the biodistribution of ANG1005 in mice bearing orthotopic human gliomas arising from intracerebral implants of U87 glioblastoma cells. Fluorescence and immunolocalisation studies demonstrated that ANG1005 and the Cy5.5Angiopep-2 accumulated in tumour tissue at significantly higher levels than in surrounding or contralateral tissue. This partitioning is most likely attributable to the presence of high LRP1 levels in individual glioblastoma cells, but may also reflect microenvironmental conditions near aggressive tumours and their influence over LRP1. The broad distribution pattern of LRP1, and its involvement in progression, suggests that ANG1005 may have broad applicability, especially in aggressive cancers, including non-brain cancers. Indeed, in patients with advanced solid tumours and brain metastases, ANG1005 therapy achieved important reductions in metastases located in a variety of organs, including the liver, lung, and lymph nodes (Sarantopoulos *et al*, 2010). In combination, these studies continue to highlight the enormous potential of EPiC chemotherapeutics for the treatment of brain tumours that, until recently, have been refractory to most available therapies.
